# Integrative Analysis of Transcriptomics and Metabolomics Reveals the Effects of Western-Style Diets on Spleen Function

**DOI:** 10.3390/biology14091136

**Published:** 2025-08-27

**Authors:** Shengguo Tang, Dongfang Li, Yanna Ma, Zhiying Zhao, Liangyuan Peng, Shuchao Liao, Haiming Ma, Hongjiang Wei

**Affiliations:** 1 Faculty of Animal Science and Technology, Yunnan Agricultural University, Kunming 650500, China; 15802587908@163.com (S.T.); dfli0927@163.com (D.L.); 2 Institute of Yunnan Circular Agricultural Industry, Pu’er 665000, China; 15874851690@163.com (Y.M.); zyzhao045@163.com (Z.Z.); 15023692717@163.com (L.P.); 17666225547@163.com (S.L.); 3 College of Animal Science and Technology, Hunan Agricultural University, Changsha 410128, China; mahaiming2000@163.com; 4 Yunnan Province Key Laboratory for Porcine Gene Editing and Xenotransplantation, Yunnan Agricultural University, Kunming 650500, China

**Keywords:** mice, spleen, immunity, transcriptomics, metabolomics

## Abstract

The spleen is essential for immunity, mediating host defense against pathogens and regulating immunological homeostasis. Western-style diets commonly cause the aggregation of body fat and the emergence of obesity. This state might lead to damage to the spleen’s functions. However, the effects of Western-style diets on gene expression and metabolic regulation in the spleen have not yet been fully explored. In this study, *C57BL/6* mice were fed either a high-fat diet (HFD) or standard chow (CHFD) for 10 weeks starting at 8 weeks old. Weekly weights were recorded, and spleens were weighed at 18 weeks. The results showed that HFD mice had significantly higher body weights from 12 weeks (*p* < 0.05) and a higher splenic index at 18 weeks (*p* < 0.01). HE staining revealed disrupted spleen structures and infarcted areas in the HFD group. Transcriptome sequencing highlighted immune-related pathways, including inflammatory response and interleukin-6 production. Among the differentially expressed genes (DEGs), *PCK1*, *ALDH9A1*, and *ALDH7A1* were significantly upregulated in the HFD group, whereas *PLA2G2F* and *PLA2G4F* exhibited significant downregulation. *APOB* emerged as a key hub gene in PPI analysis. Metabolomics analysis identified significantly different metabolites (SDMs), including Rifamycins, 7-Ketodeoxycholic Acid, Folinic Acid, and Lotaustralin, as key biomarkers for an HFD, while 1-Methylnicotinamide and Prostaglandin E1 were significant for CHFD. KEGG enrichment linked glycerophospholipid and arachidonic acid metabolism to both transcriptome and metabolome results. The joint analysis of transcriptome and metabolome data revealed that *SLC22A8* was negatively correlated with Biliverdin and 1-methylnicotinamide, and *MCPT1* was inversely correlated with 7-Ketodeoxycholic Acid. These findings offer insights into the molecular mechanisms and metabolites that influence spleen immunity and systemic immune homeostasis.

## Simple Summary

This study investigated the effects of a high-fat diet (HFD) on spleen function in C57BL/6 mice. Mice fed an HFD showed increased body weight and spleen index compared to the standard chow (CHFD) group. Histological analysis revealed structural damage in HFD spleens. Transcriptomic analysis identified changes in immune-related gene expression, with key genes such as *PCK1*, *ALDH9A1*, and *ALDH7A1* upregulated and *PLA2G2F* and *PLA2G4F* downregulated in HFD mice. Metabolomic profiling revealed distinct metabolic changes, with specific biomarkers identified for both HFD (e.g., Rifamycins, 7-Ketodeoxycholic Acid) and CHFD groups (e.g., 1-Methylnicotinamide, Prostaglandin E1). Integrated analysis of transcriptome and metabolome data highlighted key molecular pathways and metabolites, such as glycerophospholipid and arachidonic acid metabolism, that are altered by an HFD, providing insights into how Western-style diets may impact spleen immunity and systemic immune homeostasis.

## 1. Introduction

Obesity has been characterized as abnormal or excessive fat deposition [1], which may be detrimental to the health of livestock and poultry. Improper or excessive accumulation of body fat is strongly associated with the development of several diseases [2] due to persistent adipose inflammation and other disturbances to immune cell development and function [3]. At a time when the prevalence of obesity is increasing globally, an increasing number of researchers are investigating the mechanisms that induce immune dysfunction and influence the generation and treatment of disease [4].

The implications of high-fat and low-fat diets on the spleen and immune function represent a complex and important area of research [5,6,7]. The spleen is a linchpin of the immune system, filtering blood, storing immune cells, and participating in the production of antibodies [5,6]. High-fat diets typically lead to the accumulation of body fat and obesity, a state that may lead to the impairment of spleen function [7]. Research has shown that an excessive fat intake induces a chronic inflammatory response that inhibits the proliferation and activation of lymphocytes in the spleen, thereby reducing immune responsiveness [8]. In addition, a high-fat diet may also contribute to the dysfunction of macrophages in the spleen, making them less capable of clearing pathogens and increasing the risk of infection [9]. Overall, a high-fat diet may negatively affect the spleen and immune system by triggering chronic inflammation and suppressing immune cell function [10,11]. Therefore, rational control over fat intake is essential in maintaining good immune function.

The health effects of high-fat and standard chow diets represent a complex and widely researched topic, and although there are many studies in other areas on the metabolic and cardiovascular effects of high-fat and standard chow diets [12,13], the specific roles of an HFD in spleen function are still unknown. Therefore, in this study, we established a murine model of HFD and CHFD interventions and systematically characterized diet-induced changes in the spleen through the transcriptome and the metabolome, with a focus on immunologically relevant gene networks and metabolites implicated in immunological homeostasis.

## 2. Materials and Methods

### 2.1. Experimental Animals and Sample Collection

**Figure 1:**
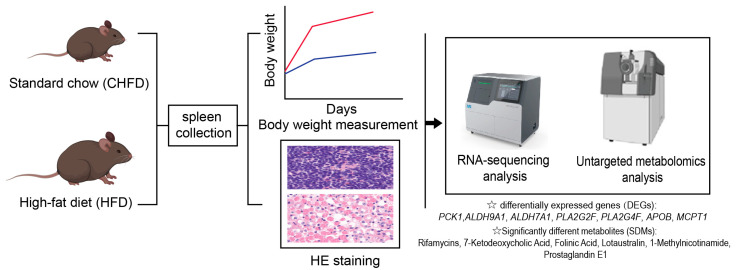
Experimental design of study.

A total of two groups (with 4 mice in each group) were established, comprising C57BL/6 mice subjected to diverse dietary regimens (Figure 1). Mice were pre-reared for one week and randomized into 2 groups (4 per group: 2 males, 2 females), and the experiments were formally conducted from the eighth week onwards. The control group was fed with a standard chow diet (CHFD group), and the HFD group was fed with a high-fat diet. Mice were maintained under controlled environmental conditions, with a 12 h light–dark cycle and a stable temperature range of 23–25 °C. Food and water were provided ad libitum. The detailed nutritional composition of the experimental diet is presented in Table S1. The feeding cycle of all experimental mice lasted for 10 weeks, and the body weight of the mice was recorded weekly. After a fasting period lasting for 15–18 h, mice were anesthetized using isoflurane, and serum was separated from blood samples extracted from the retro-orbital venous plexus. The spleen samples were weighed and collected for subsequent analysis.

### 2.2. Hematoxylin and Eosin (HE) Staining

The spleen tissues were collected and fixed in a 4% paraformaldehyde (Biosharp, Hefei, China) solution for more than 24 h. Then, the spleen tissues were dehydrated in a Leica ASP200S automatic dehydrator (Leica Biosystems, Nussloch, Germany) with gradient alcohol. The tissues were embedded in an embedding machine, and 4 µm thick sections were cut with a YD-355AT paraffin sectioning machine (Jinhua Yidi Medical Appliance Co., Ltd., Jinhua, China) and stained with hematoxylin–eosin.

### 2.3. RNA Extraction and RNA Sequencing Analysis

Total RNA was extracted from the spleen tissues using the Accurate SteadyPure Universal Genomic RNA Extraction Kit (Accurate Biology, Changsha, China) following the manufacturer’s protocols. We used 1% agarose gel to ascertain the degradation and integrity status of RNA. Moreover, the concentration was determined by using an Agilent 2100 Bioanalyzer (Agilent Technologies, Santa Clara, CA, USA). After the quality assessment, the RNA sample was used for library building and then sequencing using the Illumina platform (NovaSeq 6000, San Diego, CA, USA). When the sequencing was finished, Trim Galore (v0.4.1) was used to remove the adapter sequence and low-quality reads. Then, the filtered reports of reads were obtained from “fastp” software (v0.23.4). HISAT2 (v2.0.1) was selected to align the clean reads against the house mouse reference genome (GRCm39). The expression level of genes was quantified in fragments per kilobase of transcript per million reads (FPKM) using RSEM software (v 0.17.3) based on read counts mapped to the sequence of the genomic region. The DESeq2 package (v1.38.3) was used to identify DEGs between two groups (control vs. HFD). The assessment criteria for DEGs followed the threshold FDR < 0.05 & |log2FC| ≥ 2. Gene Ontology (GO) analysis was performed using Clusterprofiler (v4.0), and Kyoto Encyclopedia of Genes and Genomes (KEGG) enrichment analysis of DEGs was performed using KOBAS (v3.0). The threshold functional enrichment analysis was set at an FDR *p*-value < 0.05.

### 2.4. (UHPLC-MS/MS) Analysis of Spleen Sample

The extraction of metabolites was performed according to previous studies [14,15]; a 50 mg spleen sample was accurately weighed, and the metabolites were extracted using a 400 µL methanol–water (4:1, *v*/*v*) solution with 0.02 mg/mL L-2-chlorophenylalanin as an internal standard. The mixture was placed at −10 °C and then treated with a high-throughput tissue crusher (Wonbio-96c, Wanbo, Shanghai, China) at 50 Hz for 7 min, followed by ultrasound at 50 kHz for 25 min at 5 °C. The samples were left at −20 °C for 25 min to precipitate proteins. Subsequently, after centrifugation at 12,000× *g* at 4 °C for 15 min, the supernatant was carefully transferred to sample vials for further LC-MS/MS analysis.

Chromatographic separations were carried out using the UHPLC-Q Exactive HF-X system from Thermo Fisher Scientific (Waltham, MA, USA). In total, 2 μL of the supernatant sample was separated using the HSS T3 column (100 mm × 2.1 mm, 1.9 μm) before undergoing detection via mass spectrometry. The column temperature was maintained at 40 °C. The composition of the mobile phase consisted of 0.1% formic acid in water (solvent A) and 0.1% formic acid in acetonitrile (solvent B). The sample injection volume was 3 µL, and the flow rate was set to 0.5 mL/min. During the period of analysis, all samples were stored at 4 °C. ESI with negative and positive modes was used to perform MS detection of metabolites. The parameters for full-scan screening of metabolites were set as detailed below, with the voltage at −2.5 kV and 3.5 kV for negative and positive mode, respectively.

The metabolites in spleen samples were identified using ion features, including information on the fragmentation mode (the collision energy ramping from 10 to 40 eV, a fragment ion mass tolerance of 15 mDa), the retention index (RI) (retention time tolerance of ±0.2 min for database matching), and molecular weight (m/z) (mass accuracy threshold < 10 ppm, mass tolerance for database matching of 5–10 ppm) based on the HMDB (http://www.hmdb.ca/; access data: 2 May 2025) Metlin (https://metlin.scripps.edu/, access data: 2 May 2025), and Majorbio Database. Metabolites were further filtered using stringent quality control measures, including blank sample subtraction to remove background noise, signal-to-noise ratio thresholds (S/N > 3), and relative standard deviation limits (<30% in quality control samples) to ensure data reliability. Database matches were evaluated using a multi-criterion scoring system that ranked candidates based on mass accuracy, retention time alignment, and isotopic pattern consistency. The significantly different metabolites (SDMs) were identified using the criteria of a fold change ≥ 1.0 and ≤0.5, variable importance in projection (VIP) score > 1, and *p*-value < 0.05 (*t*-tests). The OPLS-DA score plot was visualized using the R package ‘ropls’ (v1.28.2). Finally, metabolic pathway identification was performed using the SDM dataset mapped to the KEGG database.

### 2.5. Quantitative Real-Time PCR (qRT-PCR) Validation

The extraction of RNA was performed according to the methods described above. Subsequently, it was subjected to reverse transcription to detect the relative mRNA levels. The comparison of expression levels for each gene was calculated using the 2^−ΔΔCt^ method on Ct values. *β-actin* was used as a housekeeping gene for normalization. The qRT-PCR was carried out in triplicate, and all primer sequences are provided in Table S2.

### 2.6. Statistical Analysis

Statistical analysis between the two groups was performed using two-way ANOVA in the R environment (v4.2.3), and data were visualized as the mean ± standard error of mean (SEM). Both R and GraphPad Prism (v9.3) software were used for data visualization.

## 3. Results

### 3.1. Differences in Growth Performance, Histomorphologic Changes in Spleen, and Overview of Transcriptomics Data

**Figure 2:**
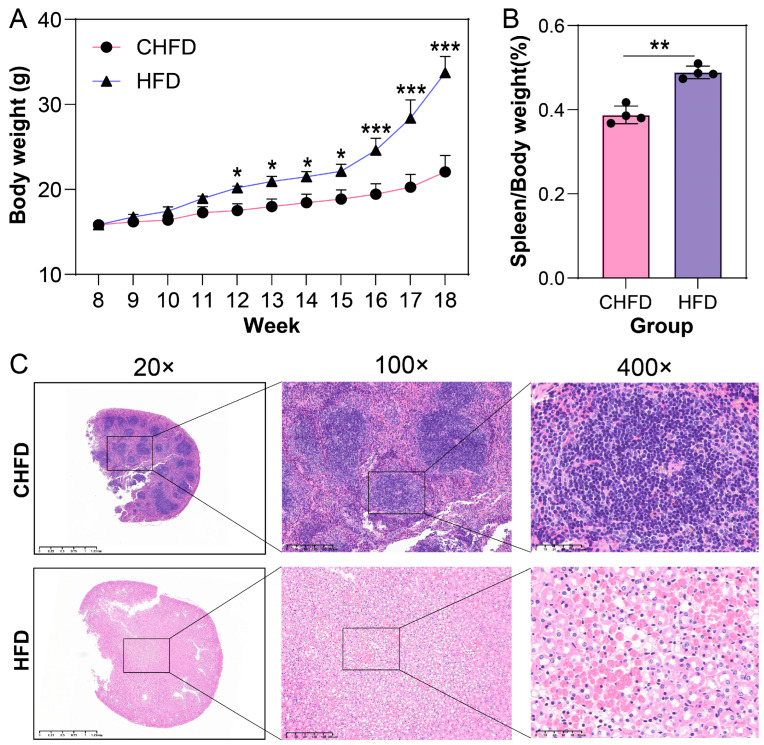
Phenotypic data of C57 mice fed with normal diet (CHFD) or high-fat diet (HFD). (**A**) Body weight (n = 4/per group); (**B**) spleen index (n = 4/per group); (**C**) HE staining of spleen. * *p* < 0.05, ** *p* < 0.01, *** *p* < 0.001.

In the present study, we raised 8-week-old mice for 10 weeks and weighed them weekly; we observed that the difference in the body weight of mice in the HFD group was significantly higher than that in the CHFD group starting from 11 weeks of age (*p* < 0.05, Figure 2A). Meanwhile, the spleen indices of the mice were calculated, and we found that the spleen indices in the HFD group were significantly higher than those in the CHFD group (*p* < 0.01, Figure 2B). HE staining of the spleen tissue revealed that the normal splenic structure was lost in the HFD group compared to the control group, the infarcted area could be observed under the high magnification of 400×, and the shadows of cells and blood vessels were still visible, with a small number of cells that had not yet been completely necrotic-scattered during the period (Figure 2C). Thus, we used transcriptome sequencing analysis to identify the key genetic or potential functional pathways in spleen tissues. In our experiments, we conducted transcriptome sequencing analysis on eight samples and obtained an average of 43.79 million clean reads and over 95.42% of Q30 bases. After obtaining clean reads, we used HISAT2 to compare them with the reference genome, and the comparison results were more than 95% (Table S3).

### 3.2. Identification of DEGs in the Spleens of Mice on Different Diets

**Figure 3:**
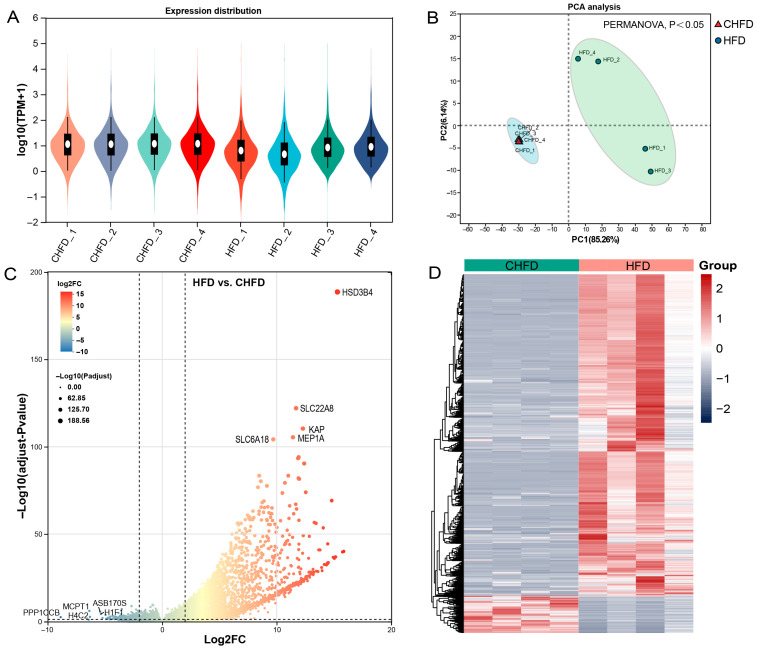
Analysis of differentially expressed genes. (**A**) Box plots of log10(TPM + 1) values for mRNA profiles (n = 4/per group). (**B**) PCA plots of all samples (n = 4/per group). (**C**) A volcano plot displaying the upregulated and downregulated DEGs. The threshold for differentially expressed genes was |log2(FC)| > 2 with an adjusted *p*-value of <0.05 (n = 4/per group). (**D**) A heatmap displaying the cluster pattern of all DEGs. HFD and CHFD indicate a high-fat diet and chow high fat diet, respectively. Upregulated features are depicted in red, and downregulated features in gray-purple in (**D**).

The box plot distributions of log10(TPM + 1) illustrate the median and quartiles of mRNA expression in the CHFD and HFD groups (Figure 3A). Principal component analysis (PCA) indicates that the different groups can be clearly distinguished (Figure 3B), which further illustrates the better quality of the RNA-seq data. Furthermore, DEG analysis was performed, and 4246 DEGs (3824 upregulated and 422 downregulated) were identified in the HFD vs. CHFD group (Figure 3C). The heatmap shows the expression patterns of the different groups (Figure 3D).

### 3.3. Transcriptome Identification of Key Regulatory Genes and Pathways in the Spleen

**Figure 4:**
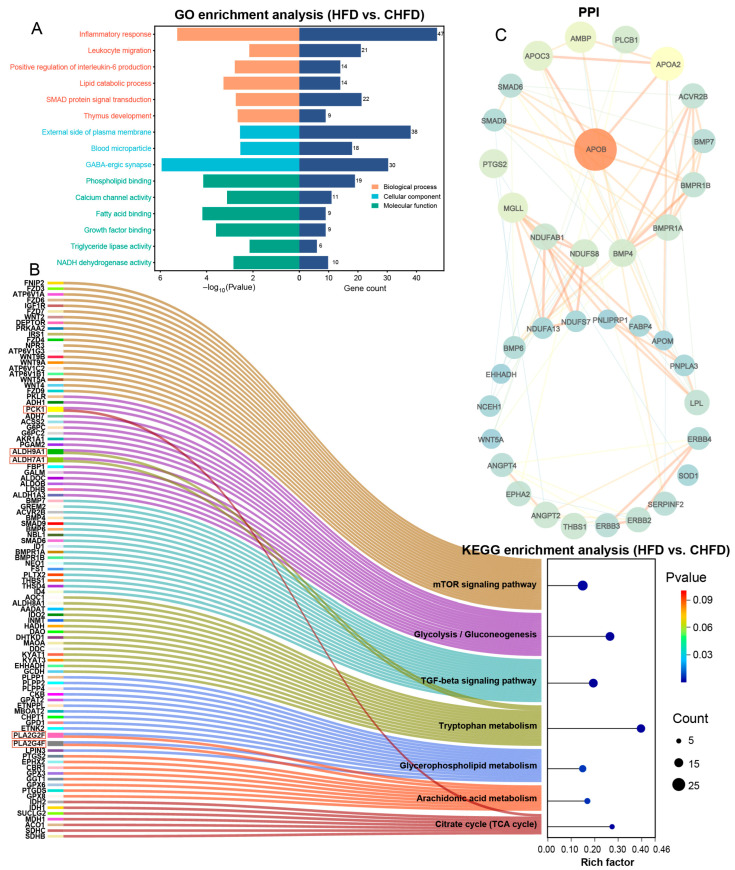
Functional analysis of transcriptome-identified DEGs. (**A**) GO analysis of DEGs; (**B**) KEGG enrichment analysis of DEGs; (**C**) PPI networks of DEGs.

To further unravel the developmental regulatory mechanisms of spleen tissues, we performed GO and KEGG functional analyses based on the dataset of DEGs. Specifically, based on an HFD vs. CHFD, GO analysis is mainly categorized into three groups: biological process (BP), cellular component (CC), and molecular function (MF). As seen in Figure 4A, inflammatory response (GO:0006954), leukocyte migration (GO:0050900), positive regulation of interleukin-6 production (GO:0032755), lipid catabolic process (GO:0016042), SMAD protein signal transduction (GO:0060395), and thymus development (GO:0048538) were enriched in the BP category. In the CC category, GABA-ergic synapse (GO:0098982) was involved. In the MF category, fatty acid binding (GO:0005504), growth factor binding (GO:0019838), and triglyceride lipase activity (GO:0004806) were annotated. KEGG enrichment analysis demonstrates (Figure 4B) that key genes (*PCK1*, *ALDH9A1*, *ALDH7A1*, *PLA2G2F*, and *PLA2G4F*) were co-enriched in the mTOR signaling pathway, glycolysis/gluconeogenesis, the TGF-beta signaling pathway, tryptophan metabolism, glycerophospholipid metabolism, arachidonic acid metabolism, and the citrate cycle (TCA cycle). The results reveal that *PCK1*, *ALDH9A1*, and *ALDH7A1* were significantly upregulated, while *PLA2G2F* and *PLA2G4F* were significantly downregulated in the HFD group. The DEGs in the regulatory network were analyzed using STRING, and the PPI network was obtained (Figure 4C), which resulted in *APOB* being the dominant hub gene and *APOB*, *APOC3*, *APOA2*, *AMBP*, *PLCB1*, *SMAD6*, *SMAD9*, *BMP4*, *BMP7*, and *BMPR1A* as the higher ranked genes.

### 3.4. Extremely Differentiated Genes and Validation of Transcriptome Sequencing Results via qRT-PCR

**Figure 5:**
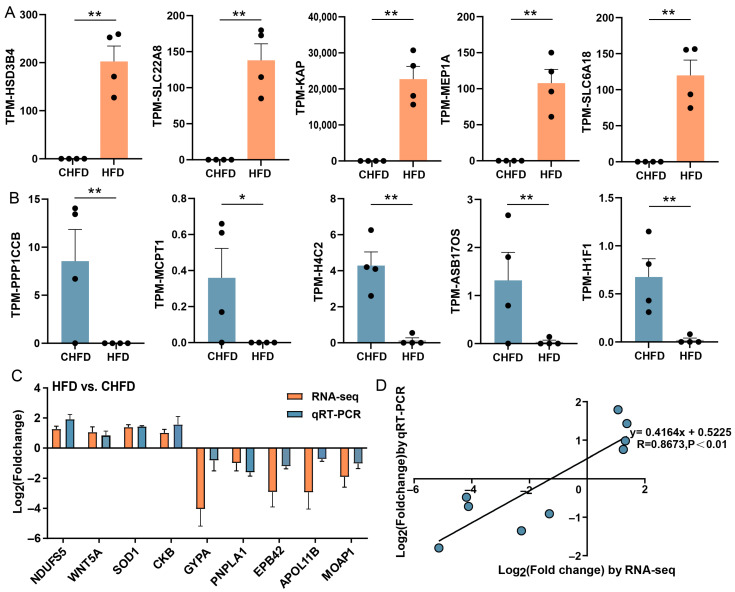
The extremity of transcriptome-identified DEGs and verification of RNA-seq data using qRT-PCR. (**A**) The top five upregulated DEGs (n = 4/per group); (**B**) the top five downregulated DEGs (n = 4/per group); (**C**) a histogram of RNA-seq and qRT-PCR expression levels of the HFD vs. CHFD group. The X-axis represents 9 selected genes, and the Y-axis represents the expression levels of genes from RNA-seq and qRT-PCR. (**D**) Linear regression analysis of expression levels between RNA-seq and qRT-PCR data. The X-axis represents the log_2_ (fold change) of RNA-seq, and the Y-axis indicates the log_2_ (fold change) of qRT-PCR. * *p* < 0.05 and ** *p* < 0.01.

Based on the filter criteria, the top five upregulated DEGs were *HSD3B4*, *SLC22A8*, *KAP*, *MEP1A*, and *SLC6A18* (Figure 5A), and the top five downregulated DEGs were *PPP1CCB*, *MCPT1*, *H4C2*, *ASB17OS*, and *H1F1* (Figure 5B). To authenticate the accuracy of the RNA-seq data, nine genes (*NDUFS5*, *WNT5A*, *SOD1*, *CKB*, *GYPA*, *PNPLA1*, *EPB42*, *APOL11B*, and *MOAP1*) were randomly selected as DEGs for qRT-PCR. The results demonstrate that the expression patterns of these genes in qRT-PCR were consistent with RNA-seq (Figure 5C), and the correlation between the two methods is relatively strenuous, with a correlation coefficient of 0.8673 (Figure 5D), indicating that the DEGs characterized via RNA-seq in this study are dependable.

### 3.5. Metabolomic Changes in the Spleen Associated with Different Diets

**Figure 6:**
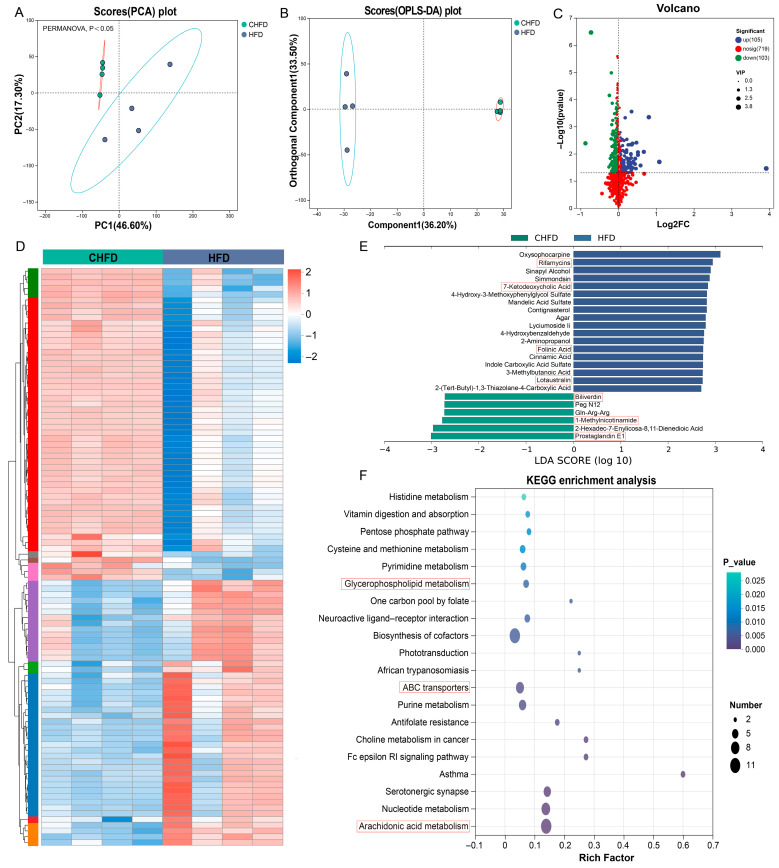
Alterations in spleen metabolomics. (**A**) PCA plots of all samples (n = 4/per group); (**B**) OPLS-DA plots of all samples (n = 4/per group); (**C**) a volcano plot displaying the upregulated and downregulated SDMs; (**D**) a heatmap displaying the cluster pattern of SDMs (n = 4/per group); (**E**) LEfSe analysis identifying the metabolic biomarkers in the spleen between the two groups. (**F**) KEGG enrichment analysis of SDMs.

To explore the metabolic changes in the spleen induced by a high-fat diet, we conducted LC-MS/MS analysis using spleen tissues. There were significant differences between the two groups, as shown by both PCA analysis and OPLS-DA analysis (Figure 6A,B). Subsequently, a total of 208 SDMs (105 upregulated and 103 downregulated) were identified based on the criteria (Figure 6C, Table S4). The heatmap demonstrates the expression patterns in the CHFD and HFD groups (Figure 6D). As a result of the LEfSe analysis (Figure 6E), we observed that metabolites of Rifamycins, 7-Ketodeoxycholic Acid, Folinic Acid, and Lotaustralin were metabolomic biomarkers for the HFD group (Figure 6E). In contrast, Biliverdin, 1-Methylnicotinamide, and Prostaglandin E1 were the major metabolites of the CHFD group. Meanwhile, our KEGG enrichment analysis revealed that three pathways, glycerophospholipid metabolism, ABC transporters, and arachidonic acid metabolism, were enriched, where glycerophospholipid metabolism and arachidonic acid metabolism overlapped with the KEGG enrichment results of DEGs (Figure 6F).

### 3.6. Association Analysis of Extreme DEGs and Key Metabolites in the Spleen

**Figure 7:**
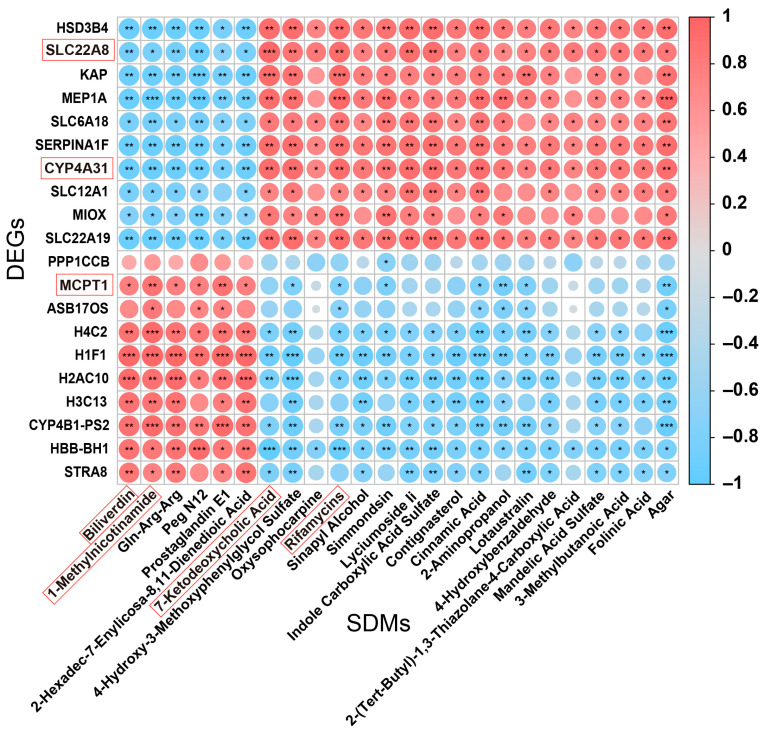
Correlation analysis between the DEGs and metabolic biomarkers. The X-axis represents SDMs, and the Y-axis represents DEGs. Spearman’s rank correlation analysis. * *p* < 0.05, ** *p* < 0.01, and *** *p* < 0.001. Red represents a positive correlation, and blue denotes a negative correlation.

We carried out a correlation analysis (Figure 7) between the top ten upregulated and downregulated genes and metabolic biomarkers. We found that DEGs, namely *SLC22A8* and *CYP4A31*, were negatively correlated with Biliverdin and 1-Methylnicotinamide, respectively. In addition, *MCPT1* was negatively correlated with 7-Ketodeoxycholic Acid and Rifamycins.

## 4. Discussion

As a key immune organ, the spleen is responsible for filtering blood, removing pathogens and senescent cells, and participating in the maturation of immune cells and the regulation of immune response [16]. A high-fat diet is known to disrupt the immunoregulatory function of the spleen, affecting the differentiation and function of T and B cells and reducing the body’s ability to mount an immune response to pathogens [17,18]. In contrast, normal feed maintains immune homeostasis of the spleen and supports the effective differentiation and functioning of immune cells, ensuring that the spleen can play its key role in pathogen clearance and immune defense [19]. Currently, there is a lack of studies on the effects of high-fat diets and normal diets on mice, especially the specific mechanisms and metabolites involved in spleen development. Therefore, we performed transcriptome analysis and metabolome analysis on the spleen to investigate the underlying biological processes affecting spleen development. Our findings may contribute to improving the molecular understanding of the possible mechanisms of the spleen and may be important in the regulation of spleen development.

We weighed the spleens of mice fed with a high-fat diet and standard chow and performed HE staining on the spleen sections. We observed that there was a significant difference between the effects of a high-fat diet and standard chow on the spleen. The high-fat diet resulted in the accumulation of body weight, an increase in the splenic index, the loss of standard splenic structure in the HFD group, and infarcted areas visualized at high magnification, whereas the standard diet contributed to the maintenance of the normal structure and functions of the spleen. Our results confirm previous findings that high-fat diets lead to the structural destruction of the spleen and decreased immune function, whereas standard diets protect the spleen through anti-inflammatory effects [20]. Hence, we further proceeded with RNA-seq to reveal the molecular mechanisms behind spleen development.

The outcome of GO enrichment analysis in this study revealed that inflammatory response, leukocyte migration, positive regulation of interleukin-6 production, lipid catabolic process, SMAD protein signal transduction, and thymus development were found to play key roles in the development of the spleen in the HFD group. Research has illustrated the effects of high-fat diets on leukocyte migration and inflammatory responses in the spleen and demonstrated that high-fat diets promote the release of inflammatory factors by altering the splenic microenvironment, thereby affecting the function and migration capacity of immune cells [21]. IL-6 impacts the function of immune cells and the intensity of inflammatory responses in the spleen by regulating the processes of lipolysis and metabolism [22]. The SMAD signaling pathway exerts a key role in thymus development and inflammatory response by regulating the expression of TGF-β family members, which consequently involves the differentiation and function of immune cells in the spleen, especially in thymus development and inflammatory response [23]. Among them, the TGF-β signaling pathway shown an association with the KEGG pathway in the current study.

In addition, KEGG analysis still identified relevant genes (*PCK1*, *ALDH9A1*, *ALDH7A1*, *PLA2G2F*, and *PLA2G4F*) involved in glycolysis/glycolysis, tryptophan metabolism, glycerophospholipid metabolism, arachidonic acid metabolism, and the citric acid cycle (TCA cycle) in the HFD group, which are engaged in functions affecting splenic development. Phosphoenolpyruvate carboxykinase 1 (*PCK1*), a rate-limiting enzyme in the regulation of gluconeogenesis, was significantly upregulated in mice with dysfunctional intestinal barriers [24], which is similar to the results of the current study. Aldehyde dehydrogenase 9 family member A1 (*ALDH9A1*) and aldehyde dehydrogenase 7 family member A1 (*ALDH7A1*) are genes of the acetaldehyde dehydrogenase (ALDH) superfamily that are involved in aldehyde detoxification, mitochondrial function, and fatty acid metabolism, as well as other cellular functions [25], and the expression of both genes was upregulated in the HFD group, consistent with the trend seen across the blood–brain barrier [26]. Both phospholipase A2 group IIF (*PLA2G2F*) and phospholipase A2 group IVF (*PLA2G4F*) modulated the release of inflammatory mediators by generating arachidonic acid [27,28,29,30]. Correspondingly, the PPI interaction network disclosed *APOB* as a core gene. *APOB* interacted in the spleen by regulating lipid metabolism [31] and influencing immune cell function and inflammatory response [32]. The upregulation of *APOB* expression significantly stimulated inflammation [33], which is in accordance with the results of this study. Meanwhile, to discover related metabolites, we proceeded to undertake a metabolomic analysis of the spleen.

The metabolomic results demonstrated that metabolites of Rifamycins, 7-Ketodeoxycholic Acid, Folinic Acid, and Lotaustralin were metabolomic biomarkers in the HFD group and that an increase in all of these metabolites represented inflammatory exacerbation [34,35,36,37]. In comparison, Cholecystokinin, 1-Methylnicotinamide, and Prostaglandin E1 were the major metabolites in the CHFD group, and the enhanced expression of these metabolites ameliorated inflammation [38,39,40]. Simultaneously, our KEGG enrichment analysis demonstrated that glycerophospholipid metabolism, ABC transporter proteins, and arachidonic acid metabolism were enriched, with glycerophospholipid metabolism and arachidonic acid metabolism overlapping with the KEGG enrichment results of RNA-seq. Furthermore, it was observed that the arachidonic acid metabolic pathway is important and that key enzymes of the arachidonic acid metabolic network, including *PLA2G2F* and *PLA2G4F*, contribute to the development of the spleen.

To further examine the top ten upregulated and downregulated genes in DEGs, they were co-analyzed with metabolic biomarkers. The results demonstrated that *SLC22A8* was negatively correlated with Biliverdin and 1-Methylnicotinamide. Moreover, *MCPT1* was negatively positively colocalized with 7-Ketodeoxycholic Acid. *SLC22A8* (organic anion transporter protein) was classified as belonging to the SLC22 family of transporter proteins, and, together with other SLC and ABC transporter proteins, it may play a pivotal role in small-molecule communication between organs and organisms [41], which may in turn affect the metabolism of Biliverdin and 1-Methylnicotinamide. There was an increase in *MCPT1* gene expression along with the induction of inflammation [42]. Here, it was also speculated that the negative correlation between *MCPT1* and 7-Ketodeoxycholic Acid would probably affect bile acid metabolism.

Derivations from the current study may provide additional insights into the specific molecular mechanisms and metabolites involved in spleen development under high-fat and standard feeds. However, there are still some limitations to our study. For example, although we mined many genes and metabolites, we did not perform the specific functional validation of specific genes or metabolites. An HFD is known to induce systemic metabolic alterations, including insulin resistance and dyslipidemia. The inclusion of additional functional assessments, such as serum lipid profiling and glucose tolerance testing, would strengthen the comprehensiveness and robustness of the dataset. Furthermore, other histological techniques, such as proteomics and lipidomics, were not applied in the current study. Therefore, it is paramount that future studies focus on these aspects.

## 5. Conclusions

In our study, the transcriptomic analysis of spleens from HFD and CHFD mice revealed enriched immune-related pathways. KEGG analysis identified several key genes (*PCK1*, *ALDH9A1*, *ALDH7A1*, *PLA2G2F*, *PLA2G4F*) involved in glycolysis, TGF-β signaling, glycerophospholipid metabolism, and arachidonic acid metabolism, crucial in splenic immune function. PPI network analysis highlighted *APOB* as a core gene. Metabolites of Rifamycins, 7-Ketodeoxycholic Acid, Folinic Acid, and Lotaustralin may play a vital role in HFD-induced spleen injury, while Cholecystokinin, 1-Methylnicotinamide, and Prostaglandin E1 may protect the spleen from the detrimental effects of an HFD. Co-analysis of top DEGs and metabolites unveiled that *SLC22A8* was negatively correlated with Biliverdin and 1-Methylnicotinamide, while *MCPT1* was inversely correlated with 7-Ketodeoxycholic Acid.

## Data Availability

The data presented in this study are available on request from the corresponding author.

## Supplementary Materials

The following supporting information can be downloaded at https://www.mdpi.com/article/10.3390/biology14091136/s1: Table S1. Nutrient components of HFD and CHFD provided to experimental mice; Table S2. Primers for real-time fluorescent quantitative PCR; Table S3. Sequencing basic data for spleen transcriptomics; Table S4. SDMs identified from comparison of HFD vs. CHFD groups.
